# Raising awareness of pre-conception care in community pharmacies: a feasibility study

**DOI:** 10.1186/s40814-022-01001-7

**Published:** 2022-02-26

**Authors:** KA Eastwood, V. A. Allen-Walker, M. Maxwell, M. C. McKinley

**Affiliations:** 1 Centre for Public Health, School of Medicine, Dentistry and Biomedical Sciences, Queen’s University Belfast, Belfast, BT12 6BJ UK; 2 Royal Jubilee Maternity Hospital, Department of Obstetrics and Gynaecology, 274 Grosvenor Road, Belfast, BT12 6BA UK; 3 Hearty Lives, Carrickfergus, Carrickfergus Borough Council, Museum and Civic Centre, Street, Carrickfergus, Antrim, BT38 7DG UK; 4 Institute for Global Food Security, Queen’s University Belfast, Belfast, BT9 5BN UK

**Keywords:** Pre-conception, Pregnancy, Intervention, Pharmacy

## Abstract

**Background:**

There is growing evidence to support the introduction of pre-conception interventions to optimise the health of mothers and their future children. At present, there is poor awareness regarding the importance of pre-conception care (PCC) amongst healthcare professionals and couples planning a pregnancy. Community pharmacies are ideally placed to reach a range of prospective couples planning a pregnancy and could effectively provide information about PCC.

**Methods:**

This study assessed feasibility of an intervention to raise awareness of PCC in community pharmacies in Northern Ireland over 3 months. Inclusion criteria: women of childbearing age (16–45 years) engaging with services at participating pharmacies. Study resources: campaign posters, information cards, crib sheets for pharmacy staff. A mixed methods approach was employed, including, brief information provision for women, record of staff interactions with customers, customer feedback cards and qualitative interviews with pharmacy staff. Descriptive statistics assessed distribution of study resources and staff interviews were analysed using a thematic analysis framework.

**Results:**

There were eight participating pharmacies, three of which consented to post-study interviews. Three pharmacies chose not to deliver the planned intervention. Distribution of campaign cards (*n* = 456) varied (0–86%). Analysis of customer feedback cards (*n* = 9) demonstrated that the majority of respondents were happy to receive information on pre-conception health. Of the women who responded to this question (*n* = 8), all were ‘extremely likely’ or ‘likely’ to act on the information provided. Four main themes emerged from analysis of staff interviews: (1) training and experience in providing health advice, (2) intervention resources, (3) understanding the aims of the intervention, (4) perceived value of the intervention. Barriers to intervention delivery included non-engagement from pharmacies and need for additional training of staff.

**Conclusions:**

An intervention to raise awareness of PCC within a community pharmacy setting was feasible and acceptable to both women and staff in participating pharmacies. This study indicates that a number of factors must be considered to enhance implementation and effectiveness of PCC interventions in this setting. In particular, better understanding of non-engagement, provision of adequate training and support for staff, and exploring incentives for pharmacies to prioritise PCC.

## Key messages regarding feasibility

- This study assessed feasibility of an intervention to raise awareness of pre-conception care in community pharmacies in Northern Ireland.
- A pre-conception care intervention within a community pharmacy setting was feasible and acceptable to both women and staff in participating pharmacies. Barriers to delivery of the intervention included non-engagement from community pharmacies and need for additional staff training.
- To enable progression to a randomised controlled trial, ongoing health promotion and additional pilot studies within different community settings are required. These should focus on exploring how to overcome key barriers identified by this research; specifically provision of adequate training and support for staff delivering pre-conception interventions, and methods to incentivise pharmacies to prioritise and actively promote pre-conception care.

## Background

Worldwide, many women planning a pregnancy are doing so against a background of increasing risk; including, maternal obesity [1], nutritional deficiency [2], advanced maternal age [3] pre-existing medical co-morbidities [4], smoking and alcohol or drug use [5]. These pre-conceptual factors put women at increased risk of antenatal, intrapartum and post-partum complications. Therefore, interventions are needed in the pre-conception period to optimise the health of mothers and their future children.

The World Health Organisation (WHO) defines pre-conception care (PCC) as “the provision of biomedical, behavioural and social health interventions to women and couples before conception occurs. Its aim is to improve maternal and child health, in both the short and long term” [6]. Yet, there is no standardised definition of the pre-conception period. Deane et al. suggest it is defined as “a minimum of one to two years prior to the initiation of any unprotected sexual intercourse that could possibly result in a pregnancy” [7]. Given the overlap with peri-conception and inter-pregnancy care, the authors extend the definition of PCC to “any intervention provided to women and couples of childbearing age, regardless of pregnancy status or desire, before pregnancy, to improve health outcomes for women, new-borns and children” [7]. Such a broad definition presents a number of challenges, including, who should have access to PCC, how they are targeted and when they should be targeted. At present, over 40% of pregnancies in the UK are unplanned; therefore, identifying those most likely to engage with PCC is important. This distinction signals both the level of responsibility assigned for changing relevant health behaviours, and the readiness of prospective parents to receive information on PCC and to act upon it [8]. Qualitative analysis exploring pre-conception health beliefs amongst adults of childbearing age in the UK has identified a willingness to learn more about pre-conception health and a need to improve pre-conception awareness within healthcare systems in a life course approach [9]. To date, the literature suggests that behavioural change is limited even in those who do plan pregnancy [10]. Therefore, efforts are required to raise awareness of PCC and the need to change lifestyle behaviours prior to conception.

Within the UK, here is increasing recognition of the need to incorporate pharmacies into the wider ‘public health workforce,’ by involvement in health promotion. This has given rise to a new concept; the Healthy Living Pharmacy (HLP) which is designed to meet public health needs and address health inequality through delivery of health and well-being services tailored to local need [11]. The Department of Health has recognised the importance of community pharmacists in delivery of healthcare interventions. In the ‘Making it better through Pharmacy in the Community’ strategy a number of key priorities are outlined for community pharmacists, including, provision of quality assured public health services and improvement of referral pathways to statutory, voluntary and community sector service providers [12]. Previous examples of health promotion within community pharmacy settings include smoking cessation, alcohol reduction, weight management, folic acid supplementation, and screening and awareness of osteoporosis [13–15]. Given their unique position at the centre of their communities, community pharmacies are well placed to introduce PCC strategies.

A number of barriers to the introduction of pre-conception interventions have been identified; many women do not consider themselves to be the target audience, PCC can be perceived as being intended only for couples who have fertility problems, [16] and women who have had children before often feel they have the required knowledge and experience of PCC [17]. Perceptions of conception as a natural event have also been cited as a barrier to the uptake of PCC, as increased emphasis on PCC may result in perceived “medicalisation” of conception [17]. Furthermore, pregnancy is often viewed as a private event, and uptake of PCC could potentially jeopardise this by making pregnancy intention public [16, 18]. For couples who do plan their pregnancies, the literature indicates that there is low awareness regarding the importance of optimising pre-conception health [19]. A cross-sectional survey of women attending three maternity services in London (*n* = 1173) revealed that although 73% of these pregnancies were planned, only 63% of women took pre-conception folic acid and less than half of women stopped smoking or reduced alcohol consumption in the pre-conception period (48% and 41%, respectively) [19].

Additional barriers to delivery of PCC exist in relation to Healthcare Practitioners (HCPs). Structured interviews conducted with a range of HCPs working in general practice, obstetrics and gynaecology, midwifery and sexual and reproductive health indicate poor awareness of pre-conception health issues [19]. Lack of knowledge and provision of training, time and resource constraints and a need for targeted public health campaigns are cited as barriers to delivery of PCC [19]. A systematic review evaluating HCPs attitudes and experience of pre-conception care revealed that most general practitioners (GPs) “feel comfortable” with providing pre-conception care, yet reported obstacles to delivery of pre-conception services including, insufficient clinical time, lack of financial reimbursement, inadequate training and lack of confidence in providing a pre-conception care service [20]. GPs also report difficulty in raising PCC with women at an opportune time, as many women present following conception [21]. Despite the reported barriers to the delivery and uptake of PCC, women do report positive attitudes towards the general concept of PCC [18] and express a desire for PCC for future pregnancies [22].

Against this background, we designed a study to examine the feasibility of an intervention to raise awareness of PCC in community pharmacies in in an urban area of Northern Ireland (NI). The study aimed to address the acceptability and feasibility of the approach by addressing the following research questions: (1) are women willing to receive information on pre-conception health from their community pharmacy? (2) Is it practical to deliver information on pre-conception health in this setting? (3) Do community pharmacy staff feel they have the time, knowledge and capability to raise awareness of pre-conception health through interactions with customers? (4) What factors should be considered if this approach was to be tested on a larger scale?

## Methods

A feasibility study was designed to facilitate development and delivery of a PCC intervention in community pharmacies in NI. Table 1 demonstrates how each of the study research questions maps on to study resources and data collection. Prior to commencing the research, the School of Medicine, Dentistry and Biomedical Sciences, Queen’s University Belfast granted ethical approval (REF: 15.26v2). The study was carried out in collaboration with Hearty Lives Carrickfergus, a British Heart Foundation funded partnership who recruited participating pharmacies via email and telephone invitation (REF: HL2011Cluster15). Between September and November 2015, implementation of the PCC intervention took place in eight community pharmacies in NI. The inclusion criteria were: women of childbearing age (16–45 years) engaging with services at participating pharmacies. Exclusion criteria: pharmacies outside the recruitment area of Carrickfergus, Greenisland and Whitehead, NI.

**Table 1:** Mapping of research questions onto study resources and data collection

Research question	Study specific resources	Data collection
1. Are women willing to receive information on pre-conception health from their community pharmacy?	• Customer feedback cards	• Monitoring distribution of campaign cards • Analysis of customer feedback cards
2. Is it practical to deliver information on pre-conception health in this setting?	• Record book	• Analysis of responses recorded in record book
3. Do community pharmacy staff feel they have the time, knowledge and capability to raise awareness of pre-conception health through interactions with customers?	• Resource development session	• Thematic analysis of qualitative interviews with pharmacy staff
4. What factors should be considered if this approach was to be tested on a larger scale?	• Campaign posters • Campaign card • Crib sheet for pharmacy staff • Staff training	• Thematic analysis of qualitative interviews with pharmacy staff

### Resource development and intervention delivery

**Fig. 1:**
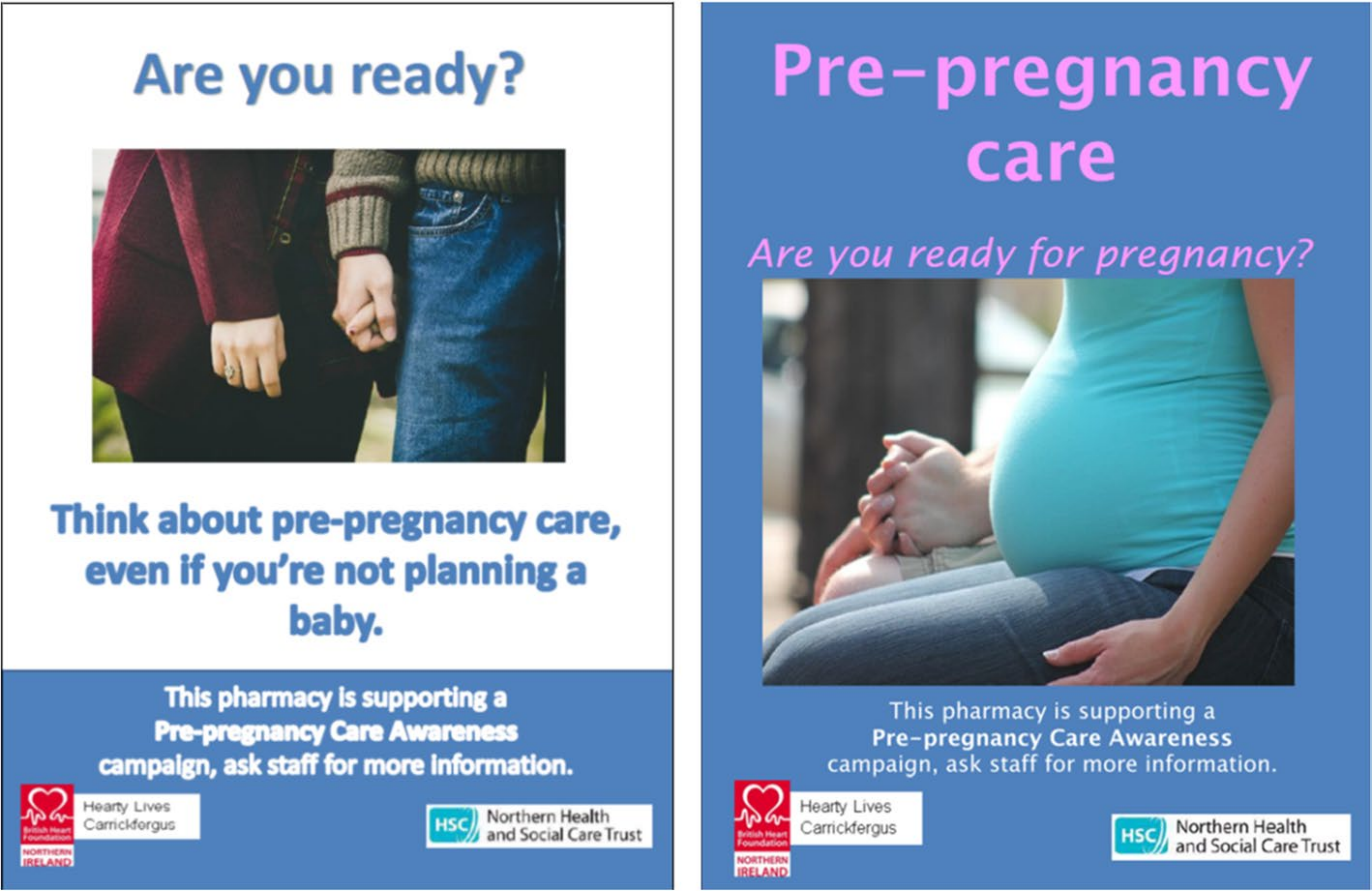
Posters advertising the pre-pregnancy awareness campaign

**Fig. 2:**
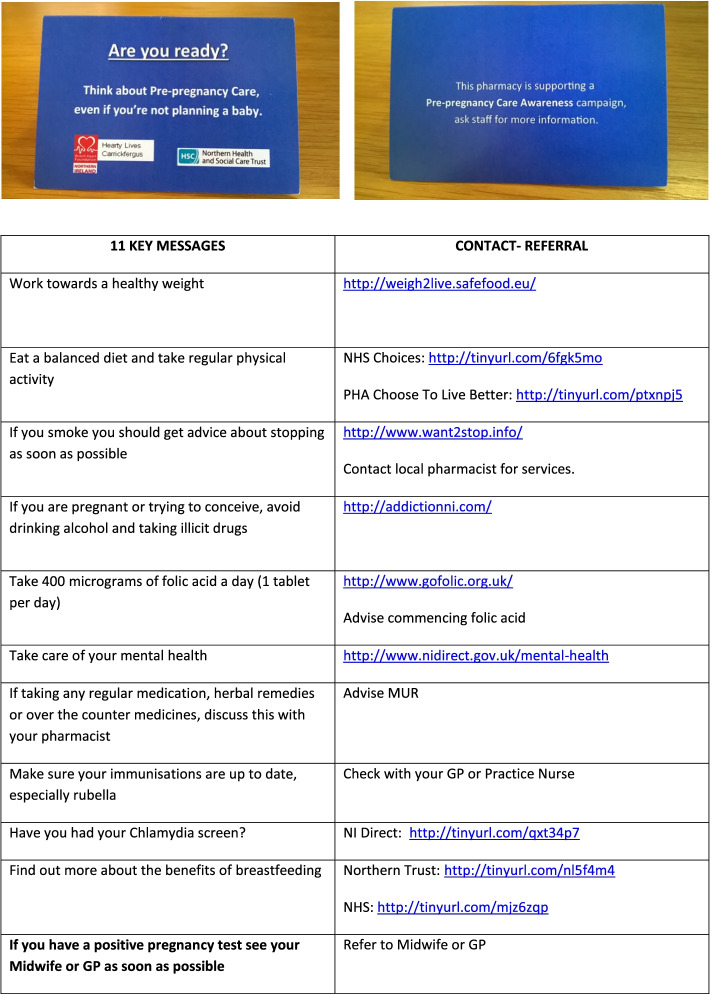
Information card for participants and key pre-conception messages (campaign credit card)

**Fig. 3:**
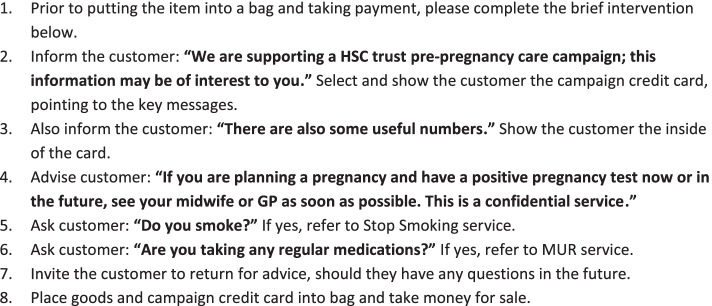
Standard operating procedure for staff delivering pre-conception care intervention

Intervention resources were developed in conjunction with staff from each of the eight participating community pharmacies. A resource development session was held over lunch time with lunch provided, to present background information for the project and gain feedback on proposed study materials. Pharmacy staff also suggested the use of posters to support the intervention. Use of appropriate terminology was also discussed; it was felt that the intervention should be described as ‘pre-pregnancy care’ rather than ‘pre-conception care,’ to increase understanding and engagement by potential participants. The following resources were subsequently developed to support the intervention and were circulated by email to pharmacies for feedback before being implemented: posters advertising the campaign (Fig. 1), a credit card-sized information card (campaign card) detailing key pre-conception health messages and signposting sources of information and local services (Fig. 2), and a crib sheet (standard operating procedure) to guide staff through the interaction with customers (Fig. 3).

Training for delivery of the intervention was provided in participating pharmacies by the study researcher and a Hearty Lives project worker with a midwifery background. Only staff present at the time of training visit received formal training from the study team. Information was given to staff about the aims of the intervention and appropriate use of the study resources. Staff were asked to display posters to advertise the campaign, place campaign cards on shop counters and provide feedback cards for women who received the intervention. In addition, the project worker visited each pharmacy between four and six times to assist staff with queries and deliver any additional information cards were necessary.

### Data collection

To monitor demand for information cards, each pharmacy was provided with 50 information cards at the beginning of the intervention and the number of cards given out to customers was recorded. Feedback cards were also provided for study participants to evaluate if customers were willing to receive information about PCC from their pharmacy, if they found the information useful, how likely they would be to act on the information received and if they had contacted any of the local services listed on the campaign credit card. Staff were encouraged to document any notable interactions with customers regarding delivery of the intervention; in particular, comments (positive or negative) from participants and their experiences of delivering a PCC intervention. Written, informed consent was given by pharmacy staff for qualitative interviews conducted following completion of recruitment. These interviews aimed to explore staff experiences of the intervention and evaluate the feasibility of delivering PCC in a community pharmacy setting.

### Data analysis

Descriptive statistics were used to assess the demand and use of study resources in participating pharmacies. Categorical responses from participant feedback cards were analysed in relation to four questions: (1) were you happy to receive information on pre-conception health from your pharmacy? (2) Did you find the information useful? (3) How likely are you to act on the information? (4) Have you contacted any of the services listed on the card? Qualitative interviews with pharmacy staff were transcribed verbatim for analysis within a thematic analysis framework [23] with an identification number assigned to each participant to ensure anonymity. A six-step process was followed to ensure appropriate identification and analysis of themes. The researcher first read and re-read the interview transcripts, initial codes were then generated, codes were then examined and potential themes were identified, preliminary themes were reviewed against the collated data and discussed with a second researcher, themes were further defined and data extracts were chosen to illustrate each theme [23].

## Results

A total of eight pharmacies from urban areas (three towns) of NI participated in the study between September and November 2015. Three pharmacies consented to post-study interviews (*n* = 3); two interviews were recorded (1R and 2R) and one interview was unrecorded (3U). Table 2 summarises provision and distribution of campaign cards across participating pharmacies during the intervention. In total, five of the participating pharmacies carried out the intervention. One pharmacy (pharmacy 5) was unable to deliver the PCC intervention and returned all cards provided. Unfortunately, due to time constraints, staff at the pharmacy were unable to participate in a follow-up interview to explore the reasons for this. Pharmacies 1 and 3 did not distribute the cards; post-study feedback suggests that the cards were misplaced during the course of the intervention. Distribution of campaign cards varied between participating pharmacies (44–86%) across the study period. Additional descriptive analysis demonstrates that overall distribution of cards increased across the intervention: in the first month distribution rates were between 20 and 70%.

**Table 2:** Distribution of campaign cards across the intervention period

Pharmacy	Total cards provided	Cards distributed (*n*,%)
1	70	0^a^
2	50	22 (44)
3	50	0^a^
4	50	29 (58)
5	50	—^b^
6	68	29 (42.6)
7	50	43 (86)
8	68	36 (52.9)

^a^Unclear if campaign cards were misplaced or distributed; staff feedback suggested non-compliance

^b^Pharmacy did not participate in the intervention

### Pharmacy staff observations

The record book to document staff experience of the intervention was poorly used. Only one entry was recorded by one pharmacy: “Patient enquired with ourselves after noticing a pre-pregnancy poster in our shop area. She was then advised on taking folic acid before the pregnancy and given relevant information.” It was therefore impossible to perform thematic analysis on this aspect of the study.

### Participant feedback cards

Nine feedback cards were received (*n* = 159 distributed, response rate 5.7%). Table 3 summarises participant responses to questions posed on feedback cards. Overall, the response to the intervention was positive; the majority of respondents (*n* = 8) reported that they were happy to receive information on pre-conception health. Of the women who gave feedback, all were ‘extremely likely’ (*n* = 4) or ‘likely’ (*n* = 4) to act on the information provided. One woman reported that she had contacted the smoking cessation service following the intervention. Comments received from these women highlighted the positive impact of the intervention: “Very useful and timely… I wasn’t aware you had to take folic acid before getting pregnant.” “I now have more awareness about prescribed medicine should I become pregnant.”

**Table 3:** Participant feedback

Question	Response	
	Yes	No
Were you happy to receive information on preconception health from your pharmacy? (*n* = 9)	8	1
Did you find the information useful? (*n* = 8)*	8	0
Have you contacted any of the services listed on the card? (*n* = 9)	1	8
How likely are you to act upon the information? (*n* = 8)^a^		
Extremely likely	4	0
Likely	4	0
Neutral	—	—
Unlikely	—	—
Extremely unlikely	—	—

^a^Response not given (*n* = 1)

### Qualitative interviews

Three post-study interviews were obtained from three different pharmacies (1R, 2R, 3U). Interview responses from 3U were noted directly onto the interview topic guide by the researcher and transcribed directly following interview. Verbal feedback obtained from other sites indicated that insufficient time was the main factor in non-engagement with post-study interviews. Thematic analysis of the transcripts identified four main themes: (1) staff felt trained and experienced in providing health advice, (2) intervention resources were effective in supporting the intervention, (3) understanding the aims of the intervention (4) perceived value of the intervention.

#### Theme 1: staff felt trained and experienced in providing health advice

Theme one suggested that staff felt that their background and training provided them with the experience necessary to deliver information about PCC: “…we’ve recently just been accredited with the health plus pharmacies…it’s basically health promotion and all that kind of stuff…it sort of comes second nature hopefully to most of the pharmacists…” (1R). One staff member also reported that they regularly received training as part of their role on different health conditions: “…you’re continually being doing training in store and things like that, and stuff coming through post, and we do modules every month and all on different topics…” (2R). The experience of, and training in health promotion in these pharmacies may in part explain their willingness to participate in the study and engage with the evaluation process. In contrast, subject 3U, a pharmacy assistant, reported that their training and experience to date was restricted to medication use only.

#### Theme 2: intervention resources were effective in supporting the intervention

Staff reported that the study resources were useful in aiding intervention delivery (theme two). The posters in particular enabled staff to initiate a conversation with customers about the intervention: “…we found people were standing, sort of reading it and so on, it gave us a bit of a lead way in as supposed to just cold calling kind of thing” (1R). The information cards were also considered convenient due to their compact size and effective in providing information about locally available services which staff could expand on when required: “…I thought the wee card was good because it’s small it would pop in your purse and that and it really had all the information…” (2R). Location and availability of services was considered a crucial part of the intervention by staff, reflecting the need to involve participating community pharmacies in resource development in order to include relevant and accessible services. Staff also found the crib sheet helpful to prompt them when delivering the intervention. One of the staff interviewed who was not involved in resource development knew that the crib sheet and record book were available but did not see these during the intervention period.

#### Theme 3: understanding the aims of the intervention

There appeared to be a difference in understanding aims of the PCC intervention (theme 3) and a number of sub themes emerged during analysis of this theme: deciding who to approach, difficulty in broaching the topic, training needs and the importance of a conversation. The inclusion criteria stated that all women of childbearing age (16–45 years) were eligible to receive the intervention, yet one staff member reported focusing on customers who were obtaining prescriptions for folic acid: “it was more like an added service to people who were getting that [folic acid] more than anything” (1R). Another staff member (3R) reported that information cards were only given out to women who purchased pregnancy tests. In contrast, one staff member demonstrated an understanding of the need to reach all women of childbearing age regardless of pregnancy intention: “…well you can still say to them because you know at the end of the day they maybe didn’t have all this information… and there’s always new things …” (2R). Difference in understanding of the intervention and willingness to promote awareness of PCC may have influenced how pharmacies decided which women to approach. This may also have impacted delivery of the intervention: “…we were maybe a wee bit limited in terms of our OTC (over the counter) medication, people don’t really ask us for folic acid we don’t do many vitamins or pregnancy tests and things like that…” (1R). One pharmacy chose not to raise the campaign verbally with women due to concerns that they might cause offence. Staff reported difficulties in broaching the topic of pre-conception care with women. Multiple reasons were given for this: challenges in identification of the target group (difficulty in correctly identifying age of women was cited as a limiting factor), difficulty in initiating conversations about PCC and concerns about intruding on women’s privacy. The difficulties highlighted by staff suggest that further training is required to support better intervention delivery, particularly for staff members who lack experience in approaching women. A further sub-theme emerging from theme 3 was the ‘importance of a conversation.’ Staff who were able to successfully build intervention delivery into a conversation reported greater ease and success with intervention delivery.

#### Theme 4: perceived value of the intervention

Theme four addressed the perceived value of the intervention. Despite the reported challenges in approaching customers, staff felt the intervention had value. They acknowledged that women seeking information about PCC often access the internet, therefore, the format of the information provided and accuracy of content was seen as beneficial: “people… are more inclined now to look things up” (2R). Staff commented on the need to raise awareness about PCC within other healthcare services, for example, general practice, to reach a wider demographic: “…in here you are pretty much limited with people because it’s just a prescription factory unfortunately…” (1R). In addition, staff highlighted the challenges of introducing PCC interventions in community settings “… in all honesty it was a way bit down the priority list” (1R).

## Discussion

### Acceptability of the intervention

This study demonstrates the acceptability of a PCC intervention in a community pharmacy setting. Although a limited number of feedback cards were received, women indicated that they were happy to receive information from their local pharmacy, with most likely or extremely likely to act on the information received. Our findings reflect previous research concerning delivery of community pharmacy interventions, including, alcohol reduction, smoking cessation, weight management, chlamydia screening, syringe exchange programmes and inoculation services [13, 24, 25]. Customer feedback also indicated that the intervention was useful, while staff interviews highlighted the current lack of information available to couples planning a pregnancy. In particular, staff underlined the ongoing need to provide PCC information for women of child-bearing age. The potential impact garnered with delivery of PCC and contraceptive services in community pharmacy settings is inestimable. A narrative review of publications on provision community pharmacist-delivered contraceptive services in the USA (*n* = 6) demonstrates the logistical ease, economic viability and patient acceptance of pharmacy led contraceptive and PCC services [26]. Di Pietro et al. previously demonstrated the ability of community pharmacies to provide PCC services through use of targeted medication reviews focusing on (1) teratogenic medications, (2) folic acid use and (3) immunisations. In a 19-week pilot phase, 1149 pharmacists working in 819 different community pharmacies were able to deliver 6602 PCC interventions [27]. There is an absence of targeted PCC interventions in community pharmacies, particularly in the UK. A review of ‘medication management services’; aimed at reducing risk of fetal exposure to teratogenic medications, in an American chain of community pharmacies, demonstrated that 731 women (36.2%) received at least one medication associated with adverse pregnancy outcome. Of these women, 74 (10.1%) had a concurrent prescription for folic acid or prenatal vitamins [28].

Analysis of the interviews undertaken with pharmacy staff supports the delivery of a PCC in a community pharmacy setting, with staff reporting that they felt trained and experienced in delivery of health promotion campaigns. Di Pietro Mager outlines the role of clinical pharmacists in fulfilling the unmet needs of women seeking pre-conception care [29]. Pharmacists are well placed to provide information regarding optimisation of pre-conception medication, including, advice on alternative non-teratogenic medications for chronic disease therapy, folic acid use and advice and support on lifestyle behaviours (smoking cessation, weight loss, alcohol intake) [29]. The previous literature in this area indicates that an expanded role for pharmacists enhances their professional status and alters public perceptions regarding the roles and responsibilities of community pharmacists [30].

Campaign posters and information cards chiefly supported implementation of the PCC. Posters proved a useful conservation starter, while the campaign cards provided reliable and appropriate information about PCC in a succinct manner. Pharmacy staff reported the direct impact of displaying posters about PCC, as some customers were prompted to ask for further information after seeing the promotional material. Of the pharmacies that participated in the intervention, overall distribution of the campaign cards was good (43–86%). A number of pharmacies also requested provision of additional cards. Poor provision of accurate and consistent PCC information and supporting materials has previously been cited as an obstacle to provision of community pharmacy-based interventions [31].

### Barriers to intervention delivery

Two main barriers influencing delivery of the intervention were identified: non-engagement from community pharmacies and need for additional training of pharmacy staff. Three pharmacies chose not to deliver the PCC intervention despite their initial participation in the study. Verbal feedback from these pharmacies indicated concern about the additional time needed to deliver the intervention. A systematic review of pharmacist and consumer views of community-based pharmacy interventions (*n* = 63 studies) highlights time pressure as a perceived barrier to delivery of a range of interventions, including, prevention of HIV/hepatitis, smoking cessation and alcohol screening [32]. In tandem, lack of awareness regarding the importance of pre-conception health amongst HCPs has previously been identified in the literature [19, 31]. The view that PCC is not yet a priority for community pharmacies was highlighted within the qualitative interviews. This could explain the lack of engagement from some of the community pharmacies. Additional explanations could include the potential conflicts involved in running a commercial business alongside delivery of healthcare interventions [33], and the need for financial incentives to ensure provision of additional services [31]. An alternative to traditional payment models are value-based incentives (VBI) which provide incentives to providers with improved outcomes [34]. This model of care has been evaluated in the USA where community pharmacies have a growing track record of improving patient health at reduced costs [35]. VBI programmes also highlight the importance of involving pharmacies in the design of the intervention, outcome measures and the VBI associated with success.

Our findings indicate that further training of pharmacy staff would be required to enhance their confidence to deliver PCC interventions. The inclusion criteria stated that all women of childbearing age (16–45 years) were eligible to receive the intervention, however, some pharmacies found it difficult to ‘identify’ these women and therefore only approached those who appeared to be planning a pregnancy; for example, those purchasing a pregnancy test or filling a prescription for folic acid. Staff reported concerns about raising the topic of PCC with eligible women due to fear of causing offence or embarrassment. Expectation of a negative reaction from customers has previously been identified as a barrier to delivery of pharmacy led interventions [36], yet not typically reported by those receiving the intervention. Selection bias amongst pharmacists is reported in other studies. Gudka et al. found that pharmacists selectively offered chlamydia screening to customers they considered ‘at risk’; rather than the agreed pre-defined cohort [24]. Similarly, analysis of semi-structured interviews undertaken with pharmacists offering chlamydia screening in London, UK felt it was more difficult to discuss the subject of chlamydia screening with customers attending for non-sexual health-related services. Consequently, many pharmacists only raised the provision of free chlamydia screening when customers were attending for emergency hormonal contraception [37]. This may help to explain why some pharmacies in our study only distributed PCC campaign cards when there was an obvious ‘linked sale.’ Additional training and support for pharmacists before and during the PCC campaign could enable them to act as advocates for raising PCC awareness. In time, this could help engender confidence in delivery of the intervention and appropriate use of supporting resources.

### Strengths and limitations

A number of strengths are apparent in this feasibility study. It was conducted in a number of local pharmacies within the target area, therefore results represent the realities faced by those delivering a PCC intervention in this community. Signposting to local resources ensured that women could access appropriate and timely services. Pharmacy staff were involved in planning the PCC intervention and developing supporting resources which aided implementation of the intervention in the planned setting. Where possible, written and verbal feedback was collected from those delivering and receiving the intervention to ascertain a wide range of views on intervention acceptability.

Limitations of this study relate to poor engagement of some community pharmacies. Three pharmacies chose not to deliver the planned intervention and this appeared to be at least partly owing to a lack of confidence from staff working in the pharmacy as discussed above; however, further exploration of barriers to engagement in PCC promotion in the pharmacy setting would be important to explore in future work. In addition, there was limited feedback from women who received the intervention and a relatively small number of staff engaged in the study evaluation interviews. As a result, a small number of views were available for analysis and only opinions from those who participated in the intervention were captured. It is possible that the experiences of intervention delivery differed between the two groups.

### Future work

Establishing a culture of ‘preparing for pregnancy’ and embedding this within wider health promotion messages is vital to the success of PCC interventions [16]. Previous studies evaluating the attitudes and behaviours of women from the Netherlands and Australia in the pre-conception period indicate that women are keen to receive pre-conception care and prioritise information about food safety, physical activity and nutritional supplementation [38–40]. At present there is limited evidence of effective ways to deliver PCC strategies in low middle income countries (LMICs) [41]. In addition, research priorities appear different from those in higher income countries. Research from the Child Health and Nutrition Research Initiative (CHNRI) suggests that LMICs seek to develop strategies to increase coverage of basic interventions, including, improved nutrition, provision of contraception and immunisation, prevention, detection and treatment of chronic and infectious diseases and reduction in exposure to harmful environmental smoke [42].

The need to include partners (male and female) in pre-conception care strategies is also recognised [43]. Partner involvement in delivery of PCC interventions has been identified as a priority by the CDC pre-conception care initiative (Centers for Disease Control and Prevention) [44]. Yet, men remain a difficult group to reach within the area of pre-conception care and little attention is focused on optimising male health prior to conception [45]. Delivery of a pre-conception care intervention within a community pharmacy setting has the potential to improve awareness of pre-conception health amongst men and access them in a unique and opportunistic manner.

## Conclusions

This feasibility study demonstrates that PCC interventions are acceptable and deliverable within the community pharmacy setting. Staff in many participating pharmacies were willing to engage with delivery of this PCC intervention, but reported time pressures and the need for additional training as barriers to successful intervention delivery. Before progressing to a larger-scale trial, pilot studies should be conducted, exploring how to overcome key barriers noted in this research; specifically, provision of adequate training and support for pharmacy staff delivering pre-conception interventions, and methods to incentivise pharmacies to prioritise and actively promote pre-conception care.

## Data Availability

The datasets used and/or analysed during the current study are available from the corresponding author on reasonable request.

## Abbreviations

CDC: Centers for Disease Control and Prevention
GPs: General practitioners
HLP: Health Living Pharmacy
LMICs: Low middle-income countries
NI: Northern Ireland
PCC: Pre-conception care
UK: United Kingdom
VBI: Value-based incentives
WHO: World Health Organisation

## References

[1] Scott-Pillai R, Spence D, Cardwell CR, Hunter A, Holmes VA (2013). The impact of body mass index on maternal and neonatal outcomes: a retrospective study in a UK obstetric population, 2004-2011. BJOG An Int J Obstet Gynaecol.

[2] Stephenson J, Heslehurst N, Hall J, Schoenaker DAJM, Hutchinson J, Cade JE (2018). Before the beginning: nutrition and lifestyle in the preconception period and its importance for future health. Lancet.

[3] Office for National Statistics. Births characteristics in England and Wales: 2017. Available from: http://www.ons.gov.uk/peoplepopulationandcommunity/birthsdeathsandmarriages/livebirths/bulletins/birthcharacteristicsinenglandandwales/2017.

[4] Knight M, Bunch K, Tuffnell D, Jayakody H, Shakespeare J, Kotnis R, Kenyon S, Kurinczuk JJ (Eds.) on behalf of MBRRACE-UK. Saving Lives, Improving Mothers’ Care - Lessons learned to inform maternity care from the UK and Ireland Confidential Enquiries into Maternal Deaths and Morbidity 2014-16. Oxford: National Perinatal Epidemiology Unit, University of Oxford; 2018.

[5] Inskip HM, Crozier SR, Godfrey KM, Borland SE, Cooper C, Robinson SM (2009). Women’s compliance with nutrition and lifestyle recommendations before pregnancy: general population cohort study. BMJ..

[6] World Health Organization (2013). Preconception care : maximizing the gains for maternal and child health.

[7] Dean SV, Lassi ZS, Imam AM, Bhutta ZA (2014). Preconception care: closing the gap in the continuum of care to accelerate improvements in maternal, newborn and child health. Reprod Health.

[8] Bonte P, Pennings G, Sterckx S (2014). Is there a moral obligation to conceive children under the best possible conditions? A preliminary framework for identifying the preconception responsibilities of potential parents. BMC Med Ethics.

[9] McGowan L, Lennon-Caughey E, Chun C, McKinley MC, Woodside JV (2020). Exploring preconception health beliefs amongst adults of childbearing age in the UK: a qualitative analysis. BMC Pregnancy Childbirth.

[10] Lum K, Sundaran R, Buck LG (2011). Evidence supporting preconception guidance. Am J Obs Gynaecol.

[11] Brown D, Portlock J, Rutter P (2012). Review of services provided by pharmacies that promote healthy living. Int J Clin Pharm.

[12] Department of Health (2018). Making it better through pharmacy in the community strategy - Progress on implementation plan.

[13] Brown TJ, Todd A, O’Malley C, Moore HJ, Husband AK, Bambra C (2016). Community pharmacy-delivered interventions for public health priorities: a systematic review of interventions for alcohol reduction, smoking cessation and weight management, including meta-analysis for smoking cessation. BMJ Open.

[14] Meijer W, Smit D, Jurgens R, de Jong-van den Berg L (2005). Improved periconceptional use of folic acid after patient education in pharmacies: promising results of a pilot study in the Netherlands. Int J Pharm Pract.

[15] Law AV, Shapiro K (2005). Impact of a community pharmacist-directed clinic in improving screening and awareness of osteoporosis. J Eval Clin Pract.

[16] Tuomainen H, Cross-Bardell L, Bhoday M, Qureshi N, Kai J (2013). Opportunities and challenges for enhancing preconception health in primary care: qualitative study with women from ethnically diverse communities. BMJ Open.

[17] Mazza D, Chapman A (2010). Improving the uptake of preconception care and periconceptional folate supplementation: what do women think?. BMC Public Health.

[18] van der Zee B, de Beaufort ID, Steegers EAP, Denktaş S (2013). Perceptions of preconception counselling among women planning a pregnancy: a qualitative study. Fam Pract.

[19] Stephenson J, Patel D, Barrett G, Howden B, Copas A, Ojukwu O (2014). How do women prepare for pregnancy? Preconception experiences of women attending antenatal services and views of health professionals. PLoS One.

[20] Steel A, Lucke J, Reid R, Adams J (2016). Review a systematic review of women’s and health professional’s attitudes and experience of preconception care service delivery. Fam Pract.

[21] Mazza D, Chapman A, Michie S (2013). Barriers to the implementation of preconception care guidelines as perceived by general practitioners: a qualitative study. BMC Health Serv Res.

[22] Goossens J, Delbaere I, Dhaenens C, Willems L, Van Hecke A, Verhaeghe S (2016). Preconception-related needs of reproductive-aged women. Midwifery..

[23] Braun V, Clarke V (2006). Using thematic analysis in psychology. Qual Res Psychol.

[24] Gudka S, Afuwape FE, Wong B, Yow XL, Anderson C, Clifford RM (2013). Chlamydia screening interventions from community pharmacies: a systematic review. Sex Health.

[25] Thomson K, Hillier-Brown F, Walton N, Bilaj M, Bambra C, Todd A (2019). The effects of community pharmacy-delivered public health interventions on population health and health inequalities: a review of reviews. Prev Med.

[26] Bright DR, DiPietro Mager NA (2019). Preconception care and contraception services: opportunities for community pharmacists. J Am Coll Clin Pharm.

[27] DiPietro Mager NA, Bright DR, Markus D, Weis L, Hartzell DM, Gartner J (2017). Use of targeted medication reviews to deliver preconception care: a demonstration project. J Am Pharm Assoc.

[28] Roath E, Bright DR, DiPietro Mager NA (2021). Retrospective evaluation of preconception care opportunities in a chain community pharmacy setting. Sci Pract Res.

[29] DiPietro Mager NA (2016). Fulfilling an unmet need: roles for clinical pharmacists in preconception care. Pharmacotherapy..

[30] Bissell P, Anderson C (2003). Supplying emergency contraception via community pharmacies in the UK: reflections on the experiences of users and providers. Soc Sci Med.

[31] Goodfellow A, Frank J, McAteer J, Rankin J (2017). Improving preconception health and care: a situation analysis. BMC Health Serv Res.

[32] Eades CE, Ferguson JS, O’Carroll RE (2011). Public health in community pharmacy: a systematic review of pharmacist and consumer views. BMC Public Health.

[33] Black K, Anderson C, Kubba A, Wellings K (2009). Involving pharmacists in sexual health research: experience from an emergency contraception study. J Fam Plan Reprod Heal Care.

[34] Pringle JL, Lee Rucker N, Domann D, Chan C, Tice B, Burns AL (2016). Applying value-based incentive models within community pharmacy practice. Am J Pharm Benefits.

[35] Pringle JL, Boyer A, Conklin MH, McCullough JW, Aldridge A (2014). The Pennsylvania project: pharmacist intervention improved medication adherence and reduced health care costs. Health Aff.

[36] Patwardhan P, Chewning B (2009). Ask, advise and refer: hypothesis generation to promote a brief tobacco-cessation intervention in community pharmacies. Int J Pharm Pr.

[37] Dabrera G, Pinson D, Whiteman S (2011). Chlamydia screening by community pharmacists: a qualitative study. J Fam Plan Reprod Heal Care.

[38] Szwajcer EM, Hiddink GJ, Maas L, Koelen MA, van Woerkum CMJ (2009). Nutrition-related information-seeking behaviours of women trying to conceive and pregnant women: evidence for the life course perspective. Fam Pract.

[39] Bookari K, Yeatman H, Williamson M (2017). Informing nutrition care in the antenatal period: pregnant women’s experiences and need for support. Biomed Res Int.

[40] Khan NN, Boyle JA, Lang AY, Harrison CL (2019). Preconception health attitudes and behaviours of women: a qualitative investigation. Nutrients..

[41] Mason E, Chandra-Mouli V, Baltag V, Christiansen C, Lassi ZS, Bhutta ZA (2014). Preconception care: advancing from “important to do and can be done” to “is being done and is making a difference”. Reprod Health.

[42] Dean S, Rudan I, Althabe F, Webb Girard A, Howson C, Langer A (2013). Setting research priorities for preconception care in low- and middle-income countries: aiming to reduce maternal and child mortality and morbidity. PLoS Med.

[43] Hemsing N, Greaves L, Poole N (2017). Preconception health care interventions: a scoping review. Sex Reprod Healthc.

[44] Centers for Disease Control and Prevention. Before pregnancy: information for men. 2018 [cited 2019 May 10]. Available from: https://www.cdc.gov/preconception/men.html

[45] Warner JN, Frey KA (2013). The well-man visit: addressing a man’s health to optimize pregnancy outcomes. J Am Board Fam Med.

